# Smartphone-Based Smoking Cessation Intervention (OKquit) for Oklahoma Tobacco Helpline Users: Protocol for a Randomized Controlled Trial

**DOI:** 10.2196/56827

**Published:** 2024-08-01

**Authors:** Michael Businelle, Jessica Becerra, Carl Witten, Sixia Chen, Krista Kezbers, Laura A Beebe, Darla E Kendzor

**Affiliations:** 1 TSET Health Promotion Research Center Stephenson Cancer Center University of Oklahoma Health Sciences Center Oklahoma City, OK United States; 2 Department of Family and Preventive Medicine University of Oklahoma Health Sciences Center Oklahoma City, OK United States; 3 Department of Biostatistics and Epidemiology Hudson College of Public Health University of Oklahoma Health Sciences Center Oklahoma City, OK United States

**Keywords:** smoking cessation, helpline, just-in-time adaptive intervention, mobile application, smartphone-based, mobile health, tobacco

## Abstract

**Background:**

Tobacco quitlines provide effective resources (eg, nicotine replacement therapy, smoking cessation counseling, and text and web-based support) for those who want to quit smoking in the United States. However, quitlines reach approximately only 1%-3% of people who smoke each year. Novel, smartphone-based, and low-burden interventions that offer 24/7 access to smoking cessation resources that are tailored to current readiness to quit may increase appeal, reach, and effectiveness of smoking cessation interventions.

**Objective:**

This study will examine the efficacy of OKquit, a low-burden smartphone-based app for smoking cessation.

**Methods:**

Approximately 500 people who smoke cigarettes and access the Oklahoma Tobacco Helpline (OTH) will be randomized to receive standard OTH care (SC) or SC plus the novel OKquit smartphone app for smoking cessation (OKquit). All participants will use a smartphone app to complete study surveys (ie, baseline, 27 weekly surveys, brief daily check-ins, and 27-week follow-up). Upon completion of daily check-ins and weekly surveys, participants will receive either trivia type messages (SC) or messages that are tailored to current readiness to quit smoking and currently experienced lapse triggers (OKquit). In addition, those assigned to receive the OKquit app will have access to on-demand smoking cessation content (eg, quit tips, smoking cessation medication tips). It is hypothesized that participants assigned to OKquit will be more likely to achieve biochemically verified 7-day point prevalence abstinence than those assigned to SC at 27 weeks post enrollment. In addition, participants who use more OTH resources (eg, more cessation coaching sessions completed) or more OKquit resources (eg, access more quit tips) will have greater biochemically verified smoking cessation rates.

**Results:**

Data collection began in September 2022 and final follow-ups are expected to be completed by May 2025.

**Conclusions:**

Data from this randomized controlled trial will determine whether the OKquit smartphone app combined with OTH care will increase smoking cessation rates over standard OTH care alone. If successful, OKquit could provide tailored intervention content at a fraction of the cost of traditional interventions. Furthermore, this type of low-burden intervention may offer a way to reach underserved populations of adults who smoke and want to quit.

**Trial Registration:**

ClinicalTrials.gov NCT05539209; https://clinicaltrials.gov/study/NCT05539209

**International Registered Report Identifier (IRRID):**

DERR1-10.2196/56827

## Introduction

Cigarette smoking continues to be the leading cause of preventable disease and death in the United States [1]. Recent estimates indicate that approximately 11.5% of adults in the United States smoke cigarettes [2]. According to the Centers for Disease Control and Prevention, smoking-related mortality accounts for as many as 480,000 deaths annually in the United States [3]. In 2004, the National Cancer Institute and the Centers for Disease Control and Prevention collaborated to establish a national telephonic portal for tobacco cessation quitlines using a toll-free number, 1-800-QUIT-NOW. This portal works by connecting callers directly with their appropriate state quitline. The effectiveness of tobacco quitlines has been well established in the literature [4-7]. In fact, both the 2008 US Public Health Service Clinical Practice Guideline and the 2020 Smoking Cessation Report of the Surgeon General endorsed state quitlines as an evidence-based method for tobacco cessation [1,8].

The Oklahoma Tobacco Helpline (OTH) was launched in 2003 to provide telephone-based tobacco cessation services to Oklahomans who want to quit smoking. The helpline is primarily funded by the Oklahoma Tobacco Settlement Endowment Trust to offer free, statewide services. OTH offers a variety of cessation services including phone counseling, automated email and text-based support, and nicotine replacement therapy (NRT, ie, nicotine patches and lozenges or gum) [9]. Over the past 2 decades, more than 490,000 Oklahoma tobacco users have received services from the program [10]. The North American Quitline Consortium has repeatedly ranked OTH as a leader in state-supported tobacco quitlines. In fact, between 2019 and 2022, the OTH had the highest reach of any US state quitline [11]. Despite its impressive ranking compared with other quitlines, OTH reaches only 3.1% of all Oklahomans who smoke annually, which leaves a large majority of Oklahomans who smoke unreached by OTH services [12].

It has been suggested that smoking cessation interventions should focus on particular difficulties that smokers are likely to encounter as they transition from being not ready to quit in the next 30 days to maintaining a successful quit attempt (eg, see Transtheoretical Model [13-15]). The Transtheoretical Model has stimulated a tremendous amount of addiction research; however, some limitations have constrained its use [16]. More recently, the Phase-Based Model (PBM) of cessation treatment has been proposed which includes four cessation phases: (1) motivation, (2) precessation, (3) cessation, and (4) maintenance [17]. The PBM suggests that specific challenges and opportunities present themselves in each phase of a quit attempt and treatments should be “phase tuned” to address these challenges and opportunities.

Smartphone-based ecological momentary assessments (EMA) are used to capture an individual’s behaviors and experiences in their natural environments [18]. EMA data have been used to tailor health behavior change interventions to an individual’s needs in real time [19-22]. For example, EMA data have been used to develop a smoking lapse risk estimator that identified 80% of all smoking lapses within 4 hours of the first lapse [23]. The Smart-Treatment (Smart-T) app offered on-demand features and used this lapse risk estimator to tailor smoking cessation messages to the current needs of socioeconomically disadvantaged adults in real time [19,20]. Research has indicated that urges to smoke, stress, and easy access to cigarettes were significantly reduced after tailored messages were delivered via the Smart-T app [24].

This randomized controlled trial aims to examine the impact of an adjunctive smartphone-based smoking cessation intervention (OKquit) combined with standard care (SC) by the OTH versus OTH care alone on biochemically verified smoking abstinence 27 weeks after study enrollment. This randomized controlled trial will also examine the impacts of OTH service engagement and use of OKquit intervention content on smoking abstinence and key lapse risk factors. To our knowledge, no study has examined whether low-burden app-based adjunctive interventions can improve state-based smoking cessation quitline quit rates.

## Methods

### Ethical Considerations

The University of Oklahoma Health Sciences Center institutional review board approved the protocol presented in this paper (reference number 14792). This trial is registered at ClinicalTrials.gov (NCT05539209). All participants provide informed consent, are notified that they can earn up to US $225 for completing study measures, and that they can withdraw from the study at any time without consequence. Data sets are deidentified via a subject identification number.

### Study Eligibility

Participants will include approximately 500 adults who smoke cigarettes and access the OTH (ie, phone or web) seeking smoking cessation treatment. Participants are eligible for the study if they meet the following criteria: (1) demonstrate interest in participating in this study by giving approval to the OTH provider to share contact details with the study research team, (2) provide proof of a valid Oklahoma residential address (eg, sending a text message of their photo ID or a photo ID along with a piece of stamped mail that was sent to them), (3) score 4 or higher on the Rapid Estimate of Adult Literacy in Medicine—Short Form (REALM-SF) [25] indicating greater than sixth-grade English literacy level, (4) are willing to quit smoking within 21 days after the randomization phone call, (5) currently smoke 5 or more cigarettes per day, (6) agree to complete daily 10- to 30-second app-prompted check-ins and 27 weekly 3- to 5-minute surveys on their personal smartphone, (7) own an active Android smartphone that is compatible with the Insight platform [26], (8) agree to install the Insight application onto their personal phone, (9) are 18 years of age and older (participant age has ranged from 18 to 80 years in similar trials with most participants reporting that they are 40-50 years of age), and (10) agree to complete the 27-week postrandomization follow-up assessment (which includes biochemical verification for those who report smoking abstinence).

### Procedure

**Figure 1:**
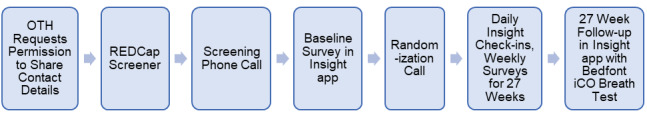
Participant flow through the OKquit study. OTH: Oklahoma Tobacco Helpline; REDCap: Research Electronic Data Capture.

Individuals who contact the OTH for smoking cessation services via the phone or web are asked whether they are interested in participating in a smoking cessation research study. Contact information for interested individuals is shared with OKquit research team members who then send a web-based Research Electronic Data Capture (REDCap) study screener link to these individuals. Those who complete the 5-minute REDCap screener and meet the initial study inclusion criteria then complete an enrollment call, which includes the informed consent process and a full study eligibility assessment. Those who enroll in the study receive instructions about how to download the Insight app onto their personal smartphone, and they are given 7 days to complete the 30-minute app-based baseline survey. Following completion of the baseline survey, research staff contact participants to randomize them into an intervention group. Randomization is stratified based on self-reported race and ethnicity (non-Hispanic White vs minoritized race and ethnicity), biological sex at birth (male vs female), and number of cigarettes smoked per day (20 vs ≥20). During the randomization call, all participants are asked to use the app to select a quit date within the next 21 days, are given instructions on how to access intervention content, and are instructed on how to complete the prompted daily check-ins and weekly surveys. Following randomization, participants interact with their assigned intervention for the 27-week study duration. Those who report abstinence from smoking during the 26th weekly survey are mailed a Bedfont carbon monoxide monitor that pairs with the Insight app by pressing the power button on the device, and they are asked to submit a breath sample for abstinence verification. Participants are also prompted to complete a 27-week follow-up assessment via the Insight app. The study flow is shown in Figure 1.

**Figure 2:**
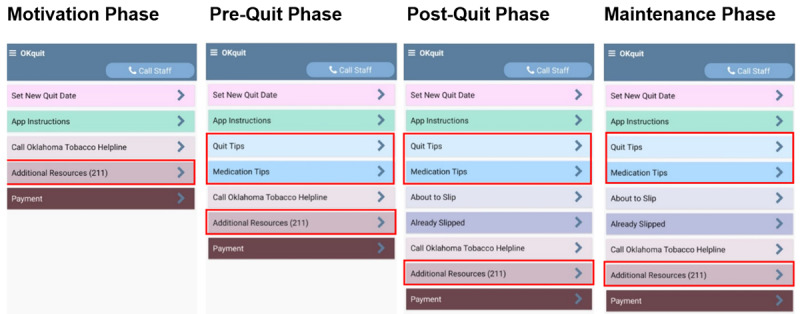
OKquit app home screen by Quit Phase. The red boxes indicate app features that are available only to the OKquit group. Features not in red boxes are available to all participants.

During the randomization call, research staff review the “App Instructions” button, which explains all app features (see Figure 2), walk participants through the schedule of daily check-ins and weekly surveys, and explain that participants will receive a US $5 for completing each weekly survey. The US $5 weekly survey is available on Saturdays via a button in the app and via app prompts. When participants do not complete the weekly survey, research staff contact them via text, call, or email to check in and remind them of the importance of completing the daily check-ins and weekly surveys. The day after randomization, the app begins to prompt a brief daily check-in 30 minutes after waking, and participants select up to 5 additional brief check-ins they would like to receive for that day. All participants are prompted to complete the daily check-ins for the entire 27-week study duration.

After initial randomization into the study and after clicking the “Set New Quit Date” button during the 27-week study period, all participants move to the Pre-–Quit Phase. Participants remain in the Pre–Quit Phase for up to 21 days (this duration is determined by the participants when they set their quit date). After setting a new quit date, the participants gain access to an app home screen that provides free smoking cessation resources including a one-click call to the OTH (both groups) and additional OKquit app resources (OKquit group only; see Figure 2). Instructions for phase-specific features are available for review at any time via the “App Instructions” button on the app home screen. During the Post–Quit Phase, the participants continue to have access to the free smoking cessation resources and additional OKquit app features through the Post–Quit Phase home screen (see Figure 2). On the morning of the quit date, the participants are prompted to complete a quit date assessment and are provided instructions about the Post–Quit Phase app features. In this phase, the participants complete up to 6 brief daily check-ins, a weekly survey, and are asked to complete slip assessments if or when they lapse. During the weekly survey, participants are asked whether they have smoked in the past 7 days. Their response to this item determines what phase they will move to next. If the participants report they have not smoked, they are asked, “How LIKELY is it that you will NOT smoke in the NEXT MONTH?” If they respond less than 8 on a 1-10 scale, they remain in the Post–Quit Phase. If they respond 8 or more, they move to the Maintenance Phase. If they report that they have returned to smoking, they are asked, “Right NOW, are you ready to get back on the wagon and set a new quit date within the next 21 days?” If they answer no to this question, they are moved to the Motivation Phase.

During the Maintenance Phase, the participants complete daily check-ins, weekly surveys, and are asked to complete slip assessments if or when they lapse. After reporting a lapse, the participants are asked whether they want to continue the quit attempt. Participants who choose to end their quit attempt are moved to the Motivation Phase. Those that choose to continue the quit attempt remain in the Maintenance Phase. In addition, all participants can click the “Set New Quit Date” button on the app home screen to change their quit date or set a new quit date at any time. Finally, during the weekly survey, the participants are asked whether they smoked within the past 7 days. If they indicate that they have smoked, they are asked whether they are ready to set a new quit date. Participants who choose a new quit date are moved back into the Pre–Quit Phase or the Post–Quit Phase. Participants move into the Motivation Phase when they report a lapse and want to end their quit attempt. Motivation Phase participants who use the “Set New Quit Date” button to set a quit date move to the Pre–Quit Phase or the Post–Quit Phase.

### Study Conditions

Those who are eligible and enroll in the study are randomly assigned to 1 of 2 conditions: Standard OTH Care (SC) or SC plus the OKquit intervention.

### Standard OTH Care (SC) Condition

Standard OTH services, including NRT (eg, nicotine patches, gum, and lozenges), phone-based counseling, and web-based intervention services are available to all participants. SC participants are prompted to complete brief daily check-ins and weekly surveys through the Insight app. In addition, SC participants have access to an on-demand one-click “Call Oklahoma Helpline” feature in their app. SC participants are able to advance through the 4 PBM phases (ie, Motivation, Pre–Quit, Post–Quit, and Maintenance); however, they do not receive tailored messages after each weekly survey or daily check-in. Instead, SC participants receive “factoid” or trivia-type messages at the completion of each check-in or weekly survey. These factoid messages serve as an attention control (see Table 1).

**Table 1:** Message types and examples.

Message level	Phase/group	Example
1	Motivation (OKquit)	First name, after quitting smoking, ex-smokers report lower levels of stress than when they were smokers.
2	Pre-Quit (OKquit)	First name, after you quit smoking, you will be tempted to lapse. Reduce temptation by removing ashtrays, lighters, matches, and cigarette butts. These items can trigger cravings and reduce motivation for staying quit.
3	Post-Quit: Low Risk for Imminent Lapse (OKquit)	First name, sometimes people save a pack of cigarettes (or even a few cigarettes) “just in case” or to prove they have the willpower to not smoke. DON’T! This just makes it easier to start smoking again!
4a: Stress	Post-Quit/Maintenance: High Risk for Imminent Lapse (OKquit)	First name, right now, it may feel like smoking would be enjoyable. However, you will feel worse later on if you do smoke now. You can get through this time without smoking.
4b: Urge	Post-Quit/Maintenance: High Risk for Imminent Lapse (OKquit)	First name, when you crave a cigarette, focus on a happy memory and get away from it all for a moment. Concentrate on that memory and nothing else.
4c: Cigarette Availability	Post-Quit/Maintenance: High Risk for Imminent Lapse (OKquit)	First name, you may be tempted to smoke when you see someone smoking or smell smoke. Do something to keep your mind off smoking. Listen to music, read, call a supportive friend, or take a walk.
4d: Cessation Motivation	Post-Quit/Maintenance: High Risk for Imminent Lapse (OKquit)	First name, you are setting a great example for children and others in your life! Congratulations on quitting smoking!
5	Maintenance: Low Risk for Imminent Lapse (OKquit)	First name, bored? Consider going to the movies or the mall—smoking is forbidden in most indoor places.
6	Post-Quit Recently Lapsed (OKquit)	First name, most smokers try to quit multiple times before they kick the habit for good, keep on trying!
7	SC^a^	It would take almost 23 million ping pong balls to fill a 747.

^a^SC: standard care.

### OKquit Condition

OKquit participants have access to all SC intervention features and app content and complete the same daily check-ins and weekly surveys as the SC group. However, OKquit participants have access to several additional features (see details in the Automated Messages and On-Demand Content sections).

#### Automated Messages

During each of the daily check-ins and weekly surveys, OKquit participants receive intervention content that is tailored to their current quitting phase (ie, Motivation, Pre-Quit, Post-Quit, and Maintenance; see Figure 2 for screenshots of the OKquit app by study phase), current level of risk for imminent lapse (ie, Cessation and Maintenance Phases only), and currently reported smoking lapse risk factors (ie, Cessation and Maintenance Phases only).

More than 900 unique messages have been embedded into the OKquit app. Messages were taken from a message bank that was developed by this research team and have been evaluated by 6 tobacco experts [27]. Table 1 provides a summary of the various message types, the phases that messages are available or used, along with example messages. All messages begin with the participant’s first name. One message is delivered at the end of each check-in and weekly survey (up to 6 per day). Level 1 messages are presented during the Motivation phase. These messages aim to increase motivation and readiness to quit smoking. Level 2 messages are presented during the Pre–Quit Phase. These messages consist of content that aims to prepare the participants for their upcoming quit attempt. During the Post–Quit and Maintenance Phases, the participants receive personalized automated messages based upon their current risk of imminent smoking (a complete description of the lapse risk estimator is presented elsewhere [23]). When participant responses indicate a low degree of risk for smoking lapse during the Post–Quit Phase, level 3 messages are presented. Level 3 messages provide general coping strategies for lapse triggers that are commonly encountered early in quit attempts. When participants report that they smoked the day prior, or they indicate that they have more than 0% likelihood of smoking that day, level 4 messages are delivered. Level 4 messages provide suggestions on ways participants could cope with 1 of 4 currently experienced smoking lapse triggers (ie, easy access to cigarettes, high stress, high urge, or low motivation for cessation). Level 5 messages are delivered during the Maintenance Phase when the app algorithms indicate low risk for imminent lapse. Level 5 messages provide general advice on sustaining longer-term abstinence. If a participant reports multiple current triggers, 1 message is delivered in this order of priority: easy access to cigarettes, high stress, high urge, or low motivation for cessation. This order is based on findings from our previous research [19,20,23,24]. Specifically, variables that are better at indicating high risk for imminent lapse are given higher priority than variables that are less strongly linked with imminent lapse. When a participant initiates a Slip assessment, a level 6 message is presented at the completion of the assessment. Level 6 messages focus on encouraging participants to resume smoking abstinence.

#### On-Demand Content

**Figure 3:**
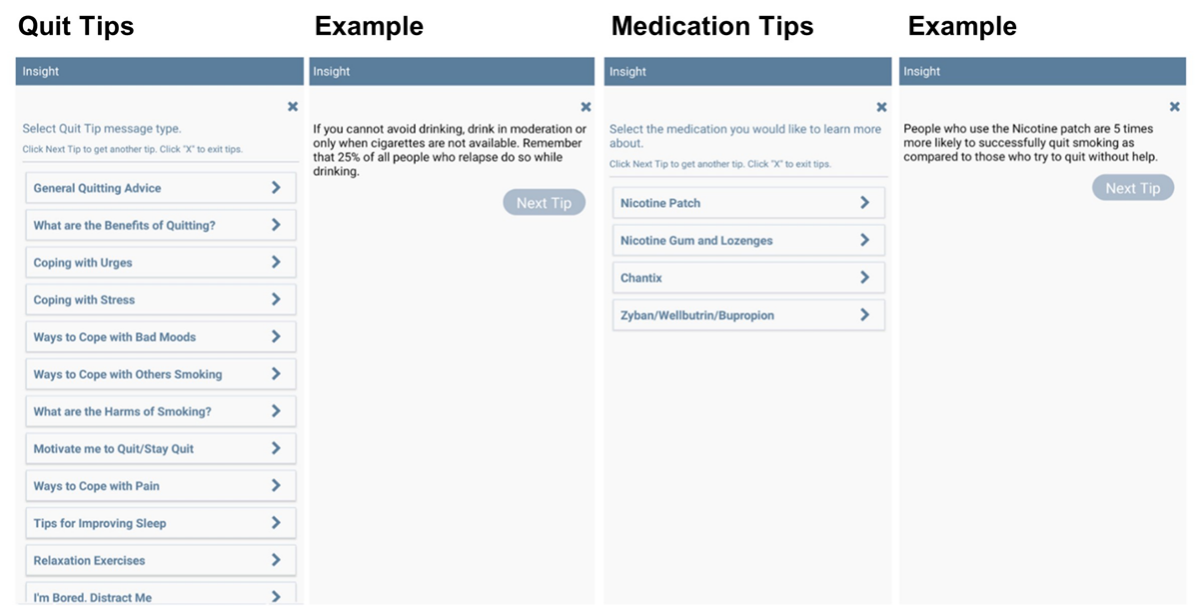
Quit Tips and Medication Tips Menus and examples available in the OKquit app.

OKquit participants have access to various on-demand features available through the smartphone app home screen. The following features are accessible 24 hours a day for 27 study weeks and include (1) the “Quit Tips” button that offers a menu of treatment-related messages, relaxation exercises, and distraction videos, (2) the “Medication Tips” button that provides information about common smoking cessation medications (eg, nicotine gum, patch, and lozenges), such as medication use tips and common medication side effects, and (3) the “Additional Resources” button that provides helpful information and website links to useful resources (eg, COVID-19 tests, food, housing, and job placement resources). When a participant clicks the “Quit Tips” or “Medication Tips” buttons, a menu of options appears (see Figure 3 for menus and example messages).

### Measures

#### Overview

The initial prescreener survey is completed via REDCap. The screener collects individual contact information and demographic information (eg, age, race, and biological sex at birth), average cigarettes smoked per day, and level of desire to quit smoking. The baseline assessment (30 minutes), weekly surveys (3-5 minutes each), daily check-ins (10-30 seconds), and 27-week follow-up assessment (20 minutes) are collected via the Insight app. See Table 2 for study assessments and administration schedule. For daily check-ins and weekly surveys, the phone rings and vibrates to prompt the participant to complete the assessment. If the participant does not respond after 5 prompts, the check-in/survey is automatically rescheduled up to 3 times 15 minutes later. Assessments collect information about several factors related to smoking, smoking lapse, and health. Participants are instructed to self-initiate “About to Slip” and “Already Slipped” assessments and to initiate the “Set New Quit Date” assessments when they want to set a new quit date. Each check-in and survey are date-, time-, and geolocation-stamped for future examination.

**Table 2:** List and timeline of study measures.

Measure	Screener	Baseline	Follow-up
Screening Questionnaire	✓		
Phone Call Screener	✓		
Rapid Estimate of Adult Literacy in Medicine—SF (REALM) [25]	✓		
Tobacco History Questionnaire		✓	✓
Heaviness of Smoking Index [28]		✓	✓
Self-Efficacy Scale/Confidence [29]		✓	✓
Demographic/Background Information Questionnaire		✓	
MacArthur Scale of Subjective Social Status [30]		✓	✓
Overall Anxiety Severity and Impairment Scale (OASIS) [31]		✓	✓
Overall Depression Severity and Impairment Scale (ODSIS) [32]		✓	✓
The Patient Health Questionnaire-4 (PHQ-4) [33]		✓	✓
Financial Strain Questionnaire [34]		✓	
Perceived Stress Scale [35]		✓	✓
The Everyday Discrimination Scale (Brief version) [36]		✓	
The Pandemic Stress Index		✓	
Perceived Social Support Questionnaire [37]		✓	
Religious Participation [38,39]		✓	✓
Self-Rated health [40]		✓	✓
Health Related Quality of Life [41]		✓	✓
Alcohol Use Disorders Identification Test (AUDIT-C) [42]		✓	✓
Marijuana/Cannabis Use Behavior		✓	✓
BRFSS Inadequate Sleep [43]		✓	✓
Short Scale of Anxiety Sensitivity Index (SSASI) [44]		✓	✓
Cannabis Use Disorder Identification Test Short Form [45]		✓	✓
Weekly EMA^a^ items		✓	✓
Smoking Status items			✓
System Usability Scale [46]			✓
Treatment Quality and Satisfaction Survey			✓

^a^EMA: ecological momentary assessments.

#### Check-Ins

Thirty minutes after the preset wake time, participants are prompted by the app to complete a morning check-in. The app welcomes participants in each condition with a different message. The SC group is told that the check-in should take less than 30 seconds to complete and given no further instructions. The OKquit group is told that the check-in should take less than 30 seconds and that their answers to the check-in questions will help the app tailor messages to their current needs. All participants are then asked, “How many cigarettes did you smoke yesterday?” OKquit participants in the Post–Quit and Maintenance Phases receive a message reminding them how many days they have been smoke-free and how much money they have saved to date by not smoking. All participants are then asked, “Which of the following apply to you RIGHT NOW? (Check all that apply).” Answer responses include the following: I could easily get cigarettes within 5-10 minutes; someone is currently smoking near me; I feel stressed; I have an urge to smoke; my motivation to quit smoking is low; I drank alcohol in the last hour; and none of the above. Next, participants are asked, “How likely is it that you will smoke between now and the end of the day?” Answer responses range from 0% to 100%. Participants are then asked, “Which of the following brief (30 seconds) check-ins would you like to schedule for today?” with the following responses: 10:00 AM, 12:00 PM, 4:00 PM, 6:00 PM, 1 hour before your usual bedtime, and None of the above. After these questions are completed, SC participants receive a level 7 (ie, factoid) message, and OKquit participants receive a message tailored to their current PBM stage and their current situation. Participants are prompted at the specified times to complete the additional check-ins that they selected for that day.

#### Weekly Survey

All participants are prompted to complete the weekly survey on Saturdays for 27 weeks. The weekly survey includes all items from the morning check-in and additional items about smoking status, current thoughts about quitting, whether they have attempted to quit smoking during the past 7 days, motivation and desire to quit smoking, cessation fatigue, alcohol and drug use, social support, and several additional items. After these questions are completed, SC participants receive a level 7 (ie, factoid) message, and OKquit participants receive a message tailored to their current PBM stage and their current situation.

#### Biochemical Verification of Smoking Status

Participants who report smoking abstinence 26 weeks after enrollment are mailed a Bedfont iCO Smokerlyzer to complete a breath test and verify their smoking status. Step-by-step instructions are presented on the smartphone screen on how to complete an iCO test. All test results are date- and time-stamped and saved for future analysis.

#### OTH Data

OTH will provide the research team with data on all interactions with study participants. In particular, the dates and number of OTH coaching sessions completed will be extracted, along with the dates and amounts of NRT ordered.

### Compensation

Participants receive compensation for completing the baseline assessment (US $20), weekly surveys (US $5 each), and the 27-week follow-up assessment (US $50) via a reloadable GreenPhire Mastercard. Participants are not compensated for completing or accessing treatment components including daily check-ins, on-demand assessments, or intervention content. Participants who report 7-day smoking abstinence at 26 weeks post enrollment (via weekly survey) and provide a breath sample are paid an additional US $20 regardless of their actual smoking status. Thus, participants may receive up to US $225 for completing all study measures. Participants can click the “Payment” button on the app home screen to review their compensation summary.

### Smartphone Programming

The mHealth Shared Resource at the NCI Designated Stephenson Cancer Center provided the smartphone app programming services for this project. This resource employs 9.5 team members including 5.5 computer scientists and engineers who develop and maintain web and mobile apps and relational databases. To date, this resource has supported 94 research studies.

### Statistical Analysis

Descriptive statistics for continuous variables will include mean, SD, and quantiles, and categorical variables will be summarized as percentages and counts. We hypothesize that participants randomly assigned to the OKquit group will have significantly higher rates of biochemically verified 7-day point prevalence smoking abstinence than those assigned to SC at 27 weeks post randomization. We will use a chi-square test or Fisher exact test to compare the proportion of participants in the treatment conditions (ie, OKquit and SC) who are biochemically verified as abstinent (ie, iCO <7 ppm, and self-reports show no smoking in the past 7 days) 27 weeks after randomization. We will conduct the analysis using the intention-to-treat assumption wherein participants who are lost to follow-up or fail to complete the iCO evaluation will be classified as not abstinent. Prior to analyzing the data, we will examine the missing rate and patterns of missing data. If the missing rate is high, then we will explore sequential multiple imputation with at least 20 imputed data files as described by Raghunathan et al [47]. The analysis results from sequential multiple imputation data will be compared with those from the available data and intention-to-treat analyses. Based upon previous studies [48], we expect that 9% of those receiving the SC intervention will be biochemically confirmed abstinent at the 27-week post–quit follow-up, and based upon our pilot work, we expect that 18% of those receiving the OKquit treatment will be abstinent at the 27-week postquit follow-up visit [19,20]. With alpha values of .05 and 250 participants in each group, we have 84% power to detect a 9% difference in biochemically confirmed 7-day point prevalence abstinence at 27 weeks (ie, 45/250, 18% abstinent in the OKquit group vs 22/250, 9% abstinent in the SC group) by using Pearson chi-square test. In addition to the chi-square test or Fisher exact analysis, we will perform logistic regression analyses to determine whether the odds of abstinence vary by other baseline characteristics (eg, sex, race, and ethnicity). Finally, we will examine interactions between treatment condition and sex and race (separately) to identify potential differential effects on abstinence.

We will examine secondary outcome measures from the collected data, including 30-day point prevalence abstinence, days to first lapse, and longest period of abstinence, which will be compared across treatment conditions. We will use the chi-square test or Fisher exact test and logistic regression models to examine 30-day point prevalence abstinence. For time to first lapse, Kaplan Meier and Cox regression modeling methods will be used. For the longest period of abstinence, *t* test or Wilcoxon rank sum test and linear regression methods will be used. Finally, we will use generalized linear mixed model regression analysis to compare abstinence rates between groups over time.

We hypothesize that OTH service engagement and use of OKquit intervention content will predict biochemically verified 7-day point prevalence smoking abstinence 27 weeks post randomization. Logistic regression models will be used to examine whether total number of OTH coaching sessions completed and OTH NRT shipments predict 27-week biochemically confirmed 7-day point prevalence abstinence. We will also examine whether the number and type of interactions with the OKquit app (eg, messages viewed) predict 27-week abstinence. Baseline characteristics such as race and sex will be considered as potential covariates during the modeling process.

## Results

The smartphone app was developed and initial data collection began in September 2022. As of July 2024, 345 participants have been enrolled. We expect to complete data collection by September 2025 and to prepare a paper describing the main outcomes by December 2025.

## Discussion

### Principal Results

This study will determine whether the OKquit app improves biochemically confirmed smoking cessation rates for OTH service users 27 weeks after enrollment. This study will also examine the effects of engagement with OTH services and OKquit intervention content on smoking abstinence and key lapse risk factors. To our knowledge, this will be the first study to examine the effects of a low-burden app-based adjunctive intervention on smoking cessation quitline quit rates.

Quitline services are evidence-based [1,8]; however, only 1%-3% of people who smoke register for quitline services each year [12,49]. Early versions of quitline services included phone counseling supplemented with mailed cessation materials. Over time, as technology has advanced, quitlines have expanded their offerings to include NRT (eg, nicotine patches, nicotine gum), web-based counseling, and mobile communications via text and email [9,10,50,51]. Adjunctive smartphone-based cessation apps such as OKquit could increase convenience, appeal, reach, and effectiveness of quitline services. Given that nearly all people in the United States own a smartphone [52], low-burden smartphone-based interventions may offer a novel way to administer empirically validated smoking cessation interventions. If using the OKquit app with and without use of standard helpline services increases biochemically verified smoking abstinence, then this type of intervention could help in the fight against smoking for individuals who might not otherwise have the means to take part in intensive smoking cessation programs. OKquit’s low-burden design includes brief daily check-ins that prompt thinking about cessation and provide useful cessation advice on how to cope with currently experienced lapse triggers. Furthermore, OKquit enables participants to access smoking cessation materials on demand and reinitiation of cessation attempts after lapse or relapse.

### Limitations

Study participants are required to use their personal Android smartphone. Individuals whose Android phone does not meet app requirements, those unwilling to install the app on their personal smartphones, those without a smartphone, and iPhone users are unable to participate. Future interventions will use a new version of the Insight platform that works with all phone types. Another limitation is that the sample will focus solely on OTH users. Finally, this study will not examine the efficacy of OKquit as a stand-alone intervention for quitline users.

### Future Work

Based upon the results of this study, future work should examine ways to improve and refine the OKquit intervention. Furthermore, future dissemination and implementation studies should examine the effectiveness of the OKquit intervention with those seeking services from smoking cessation helplines across the United States and beyond. In addition, studies should compare the effectiveness of the OKquit intervention versus standard quitline services. Finally, future work should examine the cost-effectiveness of this type of smartphone app–based intervention.

## Abbreviations

EMA: ecological momentary assessment
NRT: nicotine replacement theory
OTH: Oklahoma Tobacco Helpline
PBM: Phase-Based Model
REALM-SF: Rapid Estimate of Adult Literacy in Medicine—Short Form
REDCap: Research Electronic Data Capture
SC: standard care
Smart-T: Smart-Treatment
